# SUMO Ligase Protein Inhibitor of Activated STAT1 (PIAS1) Is a Constituent Promyelocytic Leukemia Nuclear Body Protein That Contributes to the Intrinsic Antiviral Immune Response to Herpes Simplex Virus 1

**DOI:** 10.1128/JVI.00426-16

**Published:** 2016-06-10

**Authors:** James R. Brown, Kristen L. Conn, Peter Wasson, Matthew Charman, Lily Tong, Kyle Grant, Steven McFarlane, Chris Boutell

**Affiliations:** MRC-University of Glasgow Centre for Virus Research (CVR), Garscube Campus, Glasgow, Scotland, United Kingdom; University of California, Irvine

## Abstract

Aspects of intrinsic antiviral immunity are mediated by promyelocytic leukemia nuclear body (PML-NB) constituent proteins. During herpesvirus infection, these antiviral proteins are independently recruited to nuclear domains that contain infecting viral genomes to cooperatively promote viral genome silencing. Central to the execution of this particular antiviral response is the small ubiquitin-like modifier (SUMO) signaling pathway. However, the participating SUMOylation enzymes are not fully characterized. We identify the SUMO ligase protein inhibitor of activated STAT1 (PIAS1) as a constituent PML-NB protein. We show that PIAS1 localizes at PML-NBs in a SUMO interaction motif (SIM)-dependent manner that requires SUMOylated or SUMOylation-competent PML. Following infection with herpes simplex virus 1 (HSV-1), PIAS1 is recruited to nuclear sites associated with viral genome entry in a SIM-dependent manner, consistent with the SIM-dependent recruitment mechanisms of other well-characterized PML-NB proteins. In contrast to that of Daxx and Sp100, however, the recruitment of PIAS1 is enhanced by PML. PIAS1 promotes the stable accumulation of SUMO1 at nuclear sites associated with HSV-1 genome entry, whereas the accumulation of other evaluated PML-NB proteins occurs independently of PIAS1. We show that PIAS1 cooperatively contributes to HSV-1 restriction through mechanisms that are additive to those of PML and cooperative with those of PIAS4. The antiviral mechanisms of PIAS1 are counteracted by ICP0, the HSV-1 SUMO-targeted ubiquitin ligase, which disrupts the recruitment of PIAS1 to nuclear domains that contain infecting HSV-1 genomes through mechanisms that do not directly result in PIAS1 degradation.

**IMPORTANCE** Adaptive, innate, and intrinsic immunity cooperatively and efficiently restrict the propagation of viral pathogens. Intrinsic immunity mediated by constitutively expressed cellular proteins represents the first line of intracellular defense against infection. PML-NB constituent proteins mediate aspects of intrinsic immunity to restrict herpes simplex virus 1 (HSV-1) as well as other viruses. These proteins repress viral replication through mechanisms that rely on SUMO signaling. However, the participating SUMOylation enzymes are not known. We identify the SUMO ligase PIAS1 as a constituent PML-NB antiviral protein. This finding distinguishes a SUMO ligase that may mediate signaling events important in PML-NB-mediated intrinsic immunity. Moreover, this research complements the recent identification of PIAS4 as an intrinsic antiviral factor, supporting a role for PIAS proteins as both positive and negative regulators of host immunity to virus infection.

## INTRODUCTION

Upon infection, the host mounts a coordinated immune response that restricts the replication and pathogenesis of invading viral pathogens through the combined activities of intrinsic, innate, and adaptive immunity. A key distinguishing feature of intrinsic immunity is that it is mediated by constitutively expressed cellular restriction factors that act to limit the replication and spread of many viral pathogens (reviewed in references 1 to 3). However, the mechanisms that regulate this aspect of host immunity remain to be fully elucidated.

Crucial to the intrinsic antiviral immune response during herpesvirus infection is the antiviral activity conferred by core constituent proteins associated with promyelocytic leukemia (PML) nuclear bodies (PML-NBs; also known as nuclear domain 10 [ND10]). Known restriction factors include PML (tripartite motif 19 [TRIM19]), Sp100, Daxx, and ATRX, which influence the intracellular restriction of a diverse range of viruses (4). PML, the major scaffolding protein of PML-NBs, is essential for PML-NB formation and coordinates a complex network of protein interactions dependent on sequences spanning its RBCC (RING, B-box, coiled-coil) tripartite motif (5). PML-NB formation is also heavily influenced by the posttranslational modification of PML by small ubiquitin-like modifier (SUMO) proteins (6–10), which promote noncovalent protein-protein interactions mediated by SUMO interaction motifs (SIMs) within individual PML-NB component proteins. Correspondingly, mutation of the RBCC motif, SUMO modification, or SIM consensus sequences within PML disrupts PML SUMO modification and the integrity of PML-NBs (9, 10).

SUMO modification regulates many cellular processes, including transcription, stress response, the cell cycle, and various aspects of host immunity to virus infection (reviewed in references 2 and 11). There are 3 major isoforms of SUMO (SUMO1 to SUMO3) that are conjugated within mammalian cells. SUMO2 and SUMO3 share 97% amino acid identity (and are henceforth referred to as SUMO2/3) and can form poly-SUMO chains. SUMO1 shares ∼50% amino acid identity with SUMO2 and is primarily associated with single SUMO modification or poly-SUMO chain termination events (12). Covalent attachment of SUMO to target substrates occurs in a sequential cascade analogous to that of ubiquitination, requiring E1 activating (SAE1/SAE2 heterodimer), E2 conjugating (Ubc9; also known as UBE2I), and E3 SUMO ligases (reviewed in references [Bibr B13][Bibr B14][Bibr B16]). While many SUMO modified substrates are directly conjugated by Ubc9, E3 SUMO ligases enable the selective modification of substrates in response to a wide range of stimuli, influencing aspects relating to protein-protein interaction, stability, and subcellular localization. Viruses have therefore evolved strategies to exploit or inactivate the SUMO pathway during infection in order to promote their replication (reviewed in references 2, 17, and 18).

During herpes simplex virus 1 (HSV-1) infection, SUMO modification plays a key role in the regulation of PML-NB-mediated intrinsic antiviral immunity following viral genome entry into the nucleus. SUMO modification, SUMO-SIM interactions, and a functionally active SUMO pathway all contribute to the recruitment of PML-NB-associated restriction factors to nuclear domains that contain infecting viral genomes (10, 19–21). Importantly, the recruitment of Sp100 and Daxx, as well as other SUMO2/3-conjugated proteins, to these domains occurs independently of PML (21, 22). The stable recruitment of constituent PML-NB antiviral factors correlates well with a cooperative restriction in viral gene expression (23, 24), a process that can limit the onset of productive infection and lead to the establishment of viral quiescence.

HSV-1 counteracts this aspect of host immunity through the expression of ICP0, a viral E3 ubiquitin ligase with SUMO-targeted ubiquitin ligase (STUbL) properties. ICP0 induces the proteasome-dependent degradation or dispersal of PML-NB-associated restrictions factors away from sites that contain infecting viral genomes (reviewed in references 2 and 25), as well as broadly inducing the degradation of many other SUMO-conjugated proteins (21, 26). ICP0 therefore inhibits the cellular restriction of viral gene expression and creates a favorable environment for the efficient onset of productive infection. Accordingly, ICP0-null mutant HSV-1 or HSV-1 mutants that express catalytically inactive ICP0 are more likely to enter into a nonproductive quiescent infection at low multiplicities of infection (MOI) (27–29). Importantly, the host cell restriction of these viruses can be alleviated through the depletion of PML-NB-associated restriction factors (22–24) or inactivation of the SUMO pathway through the depletion of Ubc9 (21) or PIAS4 (30), or it can be saturated upon increased MOI (25). Taken together, these data highlight a key role for the SUMO pathway in coordinating PML-NB-mediated intrinsic antiviral immunity to HSV-1 infection. However, the SUMOylation enzymes that mediate this aspect of host immunity remain to be fully defined.

We recently reported that the SUMO ligase protein inhibitor of activated STAT4 (PIAS4) contributes to the regulation of intrinsic immunity to HSV-1 infection in a manner that is cooperative with but independent of PML (30). Our identification of PIAS4 as an intrinsic antiviral factor highlights a novel role for this family of proteins that have been historically characterized as negative regulators of innate immunity (reviewed in references 15 and 16). As PIAS proteins are known to regulate innate immunity through complementary but distinct mechanisms, it is plausible that several PIAS proteins may also influence intrinsic antiviral immunity to virus infection in a PML-NB-dependent manner. Consistently, PIAS1 and PIAS2α have been reported to localize to PML-NBs and to regulate PML SUMO modification (31). We therefore investigated the potential roles of other PIAS family members in PML-NB-mediated intrinsic antiviral immunity.

We report that PIAS1 is the only family member to constitutively reside within PML-NBs in uninfected human foreskin fibroblasts (HFs) in a PIAS1-SIM and PML SUMOylation-dependent manner. During infection, PIAS1 was recruited to sites adjacent to HSV-1 genomes, also in a PIAS1 SIM-dependent manner, where it colocalized with PML-NB constituent proteins and other SUMO-conjugated proteins. In contrast to that of Daxx and Sp100 (22), the efficient recruitment of PIAS1 to these virus-induced foci was enhanced by PML. To our knowledge, this represents the first example of a PML-NB constituent protein that is recruited in a largely PML-dependent manner. The stable recruitment of PIAS1 to these foci was disrupted by ICP0 without inducing its proteasomal degradation, similar to ICP0 antagonism of Daxx (32). Depletion of PIAS1, either alone or in combination with PML or PIAS4, increased the replication efficiency of ICP0-null mutant HSV-1 but had no effect on the replication of wild-type HSV-1. Taken together, our data demonstrate that PIAS1 and PIAS4 independently contribute toward the intrinsic antiviral immune response to HSV-1 infection as part of a coordinated host response to infection that is ultimately counteracted by the E3 ubiquitin ligase activity of ICP0.

## MATERIALS AND METHODS

### Cells, drugs, and viruses.

HFt cells are immortalized human foreskin fibroblasts (HFs) (Department of Urology, University of Erlangen [22]) stably transduced with a vector that expresses the catalytic subunit of human telomerase, as described previously (33). HFs, HFt, and retinal pigmented epithelial (RPE-1; ATCC, CRL-4000) cells were maintained in Dulbecco's modified Eagle medium (DMEM; Life Technologies, 41966). Human embryonic lung (HEL 299; ECACC, 87042207) fibroblasts were maintained in minimum essential medium Eagle (MEM; Sigma-Aldrich M5650) supplemented with 2 mM l-glutamine (Life Technologies; 25030-024) and 1 mM sodium pyruvate (Life Technologies; 11360-039). HepaRG cells (34) were maintained in William's medium E (Life Technologies; 22551-022) supplemented with 2 mM l-glutamine, 5 μg/ml of insulin (Sigma-Aldrich; I2643), and 0.5 μM hydrocortisone (Sigma-Aldrich; H4881). Medium for all cell lines was supplemented with 10% fetal bovine serum (FBS; Life Technologies; 10270), 100 U/ml of penicillin, and 100 μg/ml of streptomycin (Life Technologies; 15140-122). Cells were maintained at 37°C in 5% CO_2_. HFt cells have a level of restriction of ICP0-null mutant HSV-1 replication equivalent to that of HFs (data not shown).

MG132 (Calbiochem; 474790) prepared at 10 mM in dimethyl sulfoxide (DMSO; Sigma-Aldrich; D2650) was used at 10 μM. Doxycycline (DOX; Sigma-Aldrich; D9891) prepared at 1 mg/ml in Milli-Q H_2_O was used at 0.1 μg/ml. For transgenic cells, hygromycin (Invitrogen; 10687-010), puromycin (Puro; Sigma-Aldrich; P8833), or neomycin (Neo; Sigma-Aldrich; A1720) was used at 50 μg/ml, 1 μg/ml, or 1 mg/ml, respectively, during selection or 5 μg/ml, 0.5 μg/ml, or 0.5 mg/ml, respectively, for maintenance.

Wild-type HSV-1 strain 17*syn*^+^, ICP0-null mutant HSV-1 strain *dl*1403 (27), or their respective variants that express eYFP.ICP4 (35) were used. Viruses were grown and titrated as described previously (29). When used, MG132 was added 1 h after overlay of the inoculum. For infection of inducible transgenic cell lines, enhanced yellow fluorescent protein (eYFP) or eYFP fusion protein expression was induced with DOX for 4 h prior to infection in the absence of DOX. Infected cell monolayers were then overlaid with the appropriate media supplemented with DOX for the duration of the infection.

### Plasmids and lentiviral transduction.

An eYFP-encoding sequence was PCR amplified and subcloned into the TOPO vector (Invitrogen) prior to insertion of the PIAS1 cDNA (Source Bioscience) between the EcoRI and KpnI restriction sites. The resultant eYFP.PIAS1 expression cassette was subcloned into the lentiviral vector pLKO.dCMV.TetO/R (36) using the NheI and SalI restriction sites. The primers used for PCR mutagenesis of PIAS1 and the resultant mutations are described in Table 1. All clones were confirmed by sequencing. Lentivirus vectors that express short hairpin RNAs (shRNAs) against a nontargeted control sequence (shCtrl), PML (shPML) (22), PIAS4 (shPIAS4) (30), or PIAS1 (shPIAS1; based on the sequence 5′ TTGTAAGTCGTAAGGCATGGG 3′) were obtained from the MISSION shRNA lentivirus vector collection (Sigma-Aldrich). Lentiviral supernatant stocks were produced and HFt cells were transduced as described previously (22). Transgenic cells were pooled for experimentation. Transgenic HFt cells that expressed shRNA against PML were reconstituted with PML isoform I (PML.I) or SUMOylation-deficient PML isoform I (PML.I.4KR; substitution mutations at lysine residues 65, 160, 490, and 616) as described previously (10).

**TABLE 1:** Primers for PIAS1 PCR mutagenesis and their corresponding substitution mutations

PIAS1 mutant	PCR primer for mutagenesis		Amino acid change
	Forward	Reverse	
C351A	5′GGGCCCTTACAGCGTCTCATCTAC	5′GTAGATGAGACGCTGTAAGGGCCC	C to A
mSIM	5′GAAAGTAGAAGCGGCTGACCTAACCATAG	5′CTATGGTTAGGTCAGCCGCTTCTACTTTC	VIDLT to AADLT

### Antibodies.

Primary rabbit antibodies included anti-actin (Sigma-Aldrich; A5060), anti-Daxx (Upstate; 07-471), anti-PIAS1 (LsBio; LS-B9173), anti-PIAS2 (LsBio; LS-C108717), anti-PIAS3 (LsBio; LS-C98795), anti-PIAS4 (LsBio; LS-C108719), anti-PML (Bethyl Laboratories; A301-167A), and anti-Sp100 (SpGH [37]) antibodies. Primary mouse antibodies included anti-ICP0 (11060 [38]), anti-ICP4 (58S [39]), anti-Daxx (AbD Serotec; MCA2143), anti-SUMO1 (Invitrogen; 33-2400), anti-SUMO2/3 (Abcam; ab81371), anti-UL42 (Z1F11 [40]), and anti-VP5 (DM165 [41]) antibodies. Primary sheep antibodies included anti-SUMO1 (Enzo Life Sciences; BML-PW0505) and anti-SUMO2/3 (Enzo Life Sciences; BML-PW0510) antibodies. Secondary antibodies included peroxidase-conjugated anti-mouse IgG (Sigma-Aldrich; A4416), DyLight 680- or DyLight 800-conjugated goat anti-rabbit or -mouse IgG (Thermo), and Alexa Fluor 488-, 555-, or 633-conjugated donkey anti-rabbit, -sheep, or -mouse IgG antibodies (Invitrogen).

### Immunofluorescence and confocal microscopy.

HFt cells (1 × 10^5^) seeded on 13-mm glass coverslips in 24-well plates were incubated at 37°C in 5% CO_2_ overnight. For infections, virus was diluted in serum-free DMEM to the multiplicity of infection (MOI) indicated in the corresponding figure legend. Inoculum was adsorbed for 1 h at 37°C in 5% CO_2_ with rocking and rotation every 10 min, prior to overlay with 37°C cell-appropriate media containing 2% human serum (HS; MP Biomedicals; 0929301). To examine the recruitment of host factors to nuclear sites associated with HSV-1 genome entry or replication compartments, cells were typically infected at a low MOI for 16 h in order to visualize the distribution of host factors within newly infected cells at the periphery of a developing plaque edge (20). Fixation and immunostaining were performed at room temperature. Cells were washed twice in CSK buffer (10 mM HEPES, 100 mM NaCl, 300 mM sucrose, 3 mM MgCl_2_, 5 mM EGTA), fixed and permeabilized in 1.8% formaldehyde (Sigma-Aldrich; F8775) and 0.5% Triton X-100 (Sigma-Aldrich; T-9284) in CSK buffer for 10 min, and then washed three times in CSK buffer. Cells were blocked in 2% HS in phosphate-buffered saline (PBS; Sigma-Aldrich; D1408) for 10 min, incubated with primary antibodies diluted in 2% HS in PBS for 90 min, and then washed three times in PBS. 4′,6-Diamidino-2-phenylindole (DAPI; Sigma-Aldrich; D9542) and secondary antibodies diluted 1:1,000 in 2% HS in PBS were added to the cells for 60 min, prior to three washes in PBS and two in Milli-Q H_2_O. Coverslips were then mounted on glass slides with a glycerol-based mounting medium (Citifluor; AF1) and sealed with nail enamel. Samples were examined with a Zeiss LSM 710 confocal microscope with 405-nm, 488-nm, 543-nm, and 633-nm laser lines under a Plan-Apochromat oil immersion lens, numerical aperture 1.4. Zen 2012 software (Zeiss) was used to generate cut mask channels and weighted colocalization coefficients. Three-dimensional image reconstruction was performed with Imaris (Bitplane) software. Images were minimally processed with Adobe Photoshop prior to assembly for publication with Adobe Illustrator.

### Western blotting.

HFt cells (1.5 × 10^5^ or 2.0 × 10^5^) seeded in 12-well dishes were incubated overnight at 37°C with 5% CO_2_, or for at least 4 h prior to further manipulation. For infection, 10 PFU per cell of wild-type or ICP0-null mutant HSV-1 was diluted in serum-free DMEM. Inoculum was adsorbed for 1 h at 37°C in 5% CO_2_ with rocking and rotating every 10 min, 37°C cell-appropriate medium was added, and then cells were incubated at 37°C in 5% CO_2_ until harvest. Cells were washed twice with room temperature PBS, and whole-cell lysates were collected in SDS-PAGE loading buffer with 4 M urea (Sigma-Aldrich; U0631) and 50 mM dithiothreitol (DTT; Sigma-Aldrich; D0632). Proteins resolved using standard Tris-glycine or Tris-Tricine SDS-PAGE systems were electrotransferred onto 0.2-μm nitrocellulose membranes (Amersham; 15249794) at 250 mA for 150 (Tris-Tricine) or 180 (Tris-glycine) min in Towbin buffer (25 mM Tris, 192 mM glycine, 20% [vol/vol] methanol) at room temperature. Membranes were blocked in 5% FBS in PBS overnight at 4°C. Subsequent steps were performed on a roller apparatus at room temperature. Membranes incubated with primary antibody diluted in 5% FBS in PBS with 0.1% Tween (PBST; Calbiochem; 655204) for 2 h were washed in PBST three times for 5 min each and then incubated with secondary antibody diluted 1:10,000 in 5% FBS in PBST for 1 h. Following three 5-min washes in PBST, one 5-min wash in PBS, and one rinse in Milli-Q H_2_O, membranes were imaged on an Odyssey infrared imager (Licor). The intensity of protein bands was quantified using Odyssey Image Studio software.

### Plaque forming efficiency (PFE).

HFt cells (1 × 10^5^) seeded in 24-well plates were incubated at 37°C in 5% CO_2_ overnight. Wild-type or ICP0-null mutant HSV-1 was serially diluted in serum-free DMEM. Cells inoculated with sequential viral dilutions were rocked and rotated every 10 min for 1 h and then overlaid with 37°C cell-appropriate media supplemented with 2% HS. Subsequent steps were performed at room temperature. Twenty-four hours postinfection (hpi), cells were washed twice in PBS, fixed in 1.8% formaldehyde and 0.1% NP-40 (BDH; 56009) in PBS for 10 min, and then washed in PBST twice. Cells were blocked with 5% (wt/vol) skimmed milk powder (SMP; Marvel) in PBST for 30 min and then incubated with anti-VP5 antibody diluted in 5% SMP in PBST for 90 min. Following three washes in PBST for 5 min each, peroxidase-conjugated anti-mouse IgG (Sigma-Aldrich, A4416) diluted in 5% SMP in PBST was added for 60 min. Cells were washed in PBST three times, and then plaques were visualized using True Blue peroxidase developing solution (Insight; 50-78-02) according to the manufacturer's instructions.

### Quantitative reverse transcription-PCR (RT-PCR).

A total of 1.5 × 10^5^ or 2.0 × 10^5^ HFt cells seeded in 12-well plates were incubated at 37°C in 5% CO_2_ overnight, or for at least 4 h before further manipulation. Cells were washed with PBS and total RNA was isolated using the RNAeasy Plus kit (Qiagen; 74137) or TRIzol reagent (Invitrogen; 15596) by following the manufacturer's instructions. cDNA was synthesized using the TaqMan reverse transcription reagent kit (Life Technologies; N8080234) with oligo(dT) primers. Samples were analyzed in triplicate using TaqMan Fast Universal PCR master mix (Life Technologies; 4352042) with TaqMan gene-specific primer (6-carboxyfluorescein [FAM]/MGB)-probe mixes (Life Technologies) for the following: PML (Hs00231241_m1), PIAS1 (Hs00184008_m1), 18S (GenBank accession number X03205.1), or glyceraldehyde-3-phosphate dehydrogenase (GAPDH; Life Technologies product code 4333764F).

## RESULTS

### PIAS1 is a constituent PML-NB protein.

**FIG 1:**
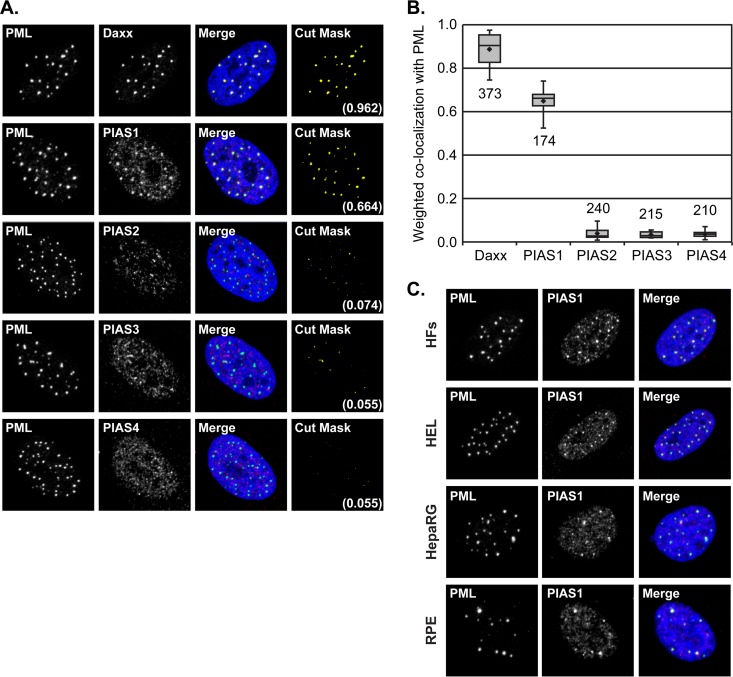
PIAS1 is a PML-NB constituent protein. (A) Confocal microscopy images show the nuclear localization of PIAS1-4 with respect to PML, the major PML-NB scaffolding protein, in HFt cells. The localization of Daxx, a major PML-NB constituent protein, with respect to PML is shown for comparison. PIAS1-4 (red), Daxx (red), and PML (green) were visualized by indirect immunofluorescence. Cut mask (yellow) highlights regions of colocalization; weighted colocalization coefficients are indicated. (B) Box-and-whisker plot showing the distribution of individual weighted colocalization coefficients of Daxx or PIAS1 to -4 with PML. The number of nuclei evaluated for each pairwise comparison is shown. Boxes, upper to lower quartiles; diamonds, means; horizontal lines, medians; upper whiskers, maximum values; lower whiskers, minimum values. (C) Confocal microscopy images show the nuclear localization of PIAS1 and PML in HFs, HEL, HepaRG, or RPE cells. PIAS1 (red) and PML (green) were visualized by indirect immunofluorescence. Nuclei were stained with DAPI (blue).

Although PML-NB-mediated restriction of viral replication is executed in part through SUMO-dependent processes (10, 21), the specific contributions of SUMOylation enzymes to this aspect of intrinsic immunity are not fully known. Using HFt human fibroblasts, which provide an amenable system to study PML-NB-mediated intrinsic immunity, we recently determined that the SUMO E3 ligase PIAS4 is an intrinsic antiviral factor. Even though PIAS4 is not a major PML-NB constituent protein in HFt cells (30), it is possible that several PIAS proteins confer intrinsic antiviral activity and that some of these proteins could regulate PML-NB-mediated viral restriction. To initiate investigation into the potential roles of PIAS proteins in PML-NB-mediated intrinsic immunity, whether any PIAS protein is a prominent constituent of PML-NBs in HFt cells was evaluated. PIAS proteins were predominantly nuclear in HFt cells and largely associated with matrix-associated regions (MARs) (Fig. 1A) (30). PIAS1 was the only family member that accumulated in punctate structures, which were identified to be PML-NBs by colocalization with PML (Fig. 1A) (30, 31). This localization of PIAS1 at PML-NBs was not unique to HFt cells and was evident in several other evaluated cell lines, including nonimmortalized fibroblasts (HFs and HEL cells), hepatocytes (HepaRG), and epithelial cells (RPE cells) (Fig. 1C). The colocalization of PIAS1 with PML within HFt cells was nearly as prominent as that of Daxx, a major constituent protein of PML-NBs (Fig. 1A and B) (42). The average weighted coefficient for colocalization of Daxx and PML was 0.89, whereas that of PIAS1 and PML was 0.65 (Fig. 1B). In contrast, PIAS2, PIAS3, and PIAS4 had minimal, if any, colocalization with PML (Fig. 1A and B). These data identify PIAS1 as the only PIAS protein that substantially localizes to PML-NBs in HFt cells.

### The localization of PIAS1 at PML-NBs is SIM-dependent and requires SUMOylated PML.

**FIG 2:**
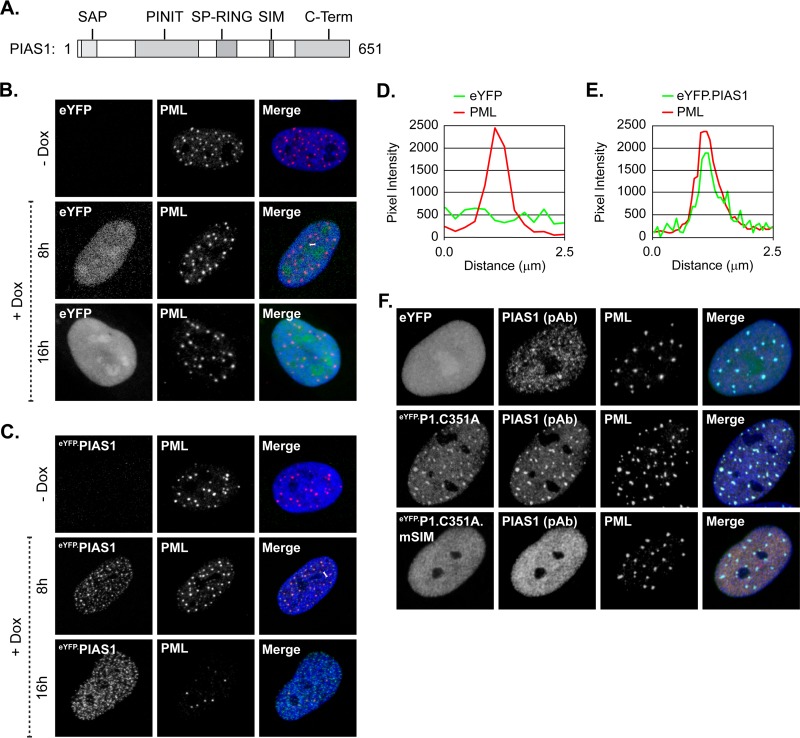
Ectopic PIAS1 associates with and disrupts PML-NBs in a SIM- and SUMO ligase-dependent manner. (A) Diagram highlighting the PIAS family conserved functional domains (gray) within PIAS1. SAP, SAF-A/B, Acinus, and PIAS; PINIT, “PINIT” motif; SP-RING, Siz/PIAS-RING zinc finger; SIM, SUMO interaction motif; C-Term, variable C-terminal domain. (B and C) Confocal microscopy images show the nuclear localization of eYFP (B) or eYFP-PIAS1 (C) with respect to PML. EYFP or eYFP-PIAS1 expression was DOX induced (+Dox), or not (−Dox), for 8 or 16 h. PML (red) was visualized by indirect immunofluorescence. (D and E) Emission spectra show the pixel intensity and colocalization between PML and eYFP (D) or eYFP-PIAS1 (E) in the regions indicated by white bars in panel B or C, respectively. (F) Confocal microscopy images show the nuclear localization of eYFP or catalytically inactive eYFP-PIAS1, with (eYFP.P1.C351A.mSIM) or without (eYFP.P1.C351A) a mutant SIM, with respect to PML-NBs. Expression of eYFP or eYFP-PIAS1 mutant proteins was DOX induced for 16 h. PML-NBs were identified by the accumulation of PML. PIAS1 (pAb; red) and PML (cyan) were visualized by indirect immunofluorescence. Nuclei were stained with DAPI (blue). pAb, polyclonal antibody.

SUMO-SIM interactions are vital for the localization of many constituent proteins at PML-NBs in uninfected cells (7, 8, 43). The role of SUMO-SIM interactions to mediate the steady-state association of PIAS1 with PML-NBs was therefore evaluated. HFt cells were stably transduced with lentiviral vectors that could be induced to express eYFP or eYFP fused to wild-type PIAS1 or PIAS1 with point mutations in its SIM domain (Fig. 2A; Table 1). EYFP did not localize at PML-NBs (Fig. 2B and D), nor did fusion to eYFP adversely affect PIAS1 localization at PML-NBs (Fig. 2C and E). Following short periods of induction, PIAS1 colocalized with PML-NBs; however, the sustained ectopic expression of PIAS1 (16 h) disrupted them (Fig. 2C) (30, 31). To avoid such adverse effects, catalytically inactive PIAS1 (eYFP.P1.C351A [Table 1]) was subsequently used (44). Inactivation of the PIAS1 SUMO ligase activity did not abrogate PIAS1 localization at PML-NBs, nor did expression of this mutant disrupt PML-NBs (Fig. 2F, middle row). Additional mutation of the SIM abrogated the localization of PIAS1 at PML-NBs (Fig. 2F, bottom row), demonstrating that PIAS1 localizes to PML-NBs in a SIM-dependent manner that is largely independent of its SUMO ligase activity.

**FIG 3:**
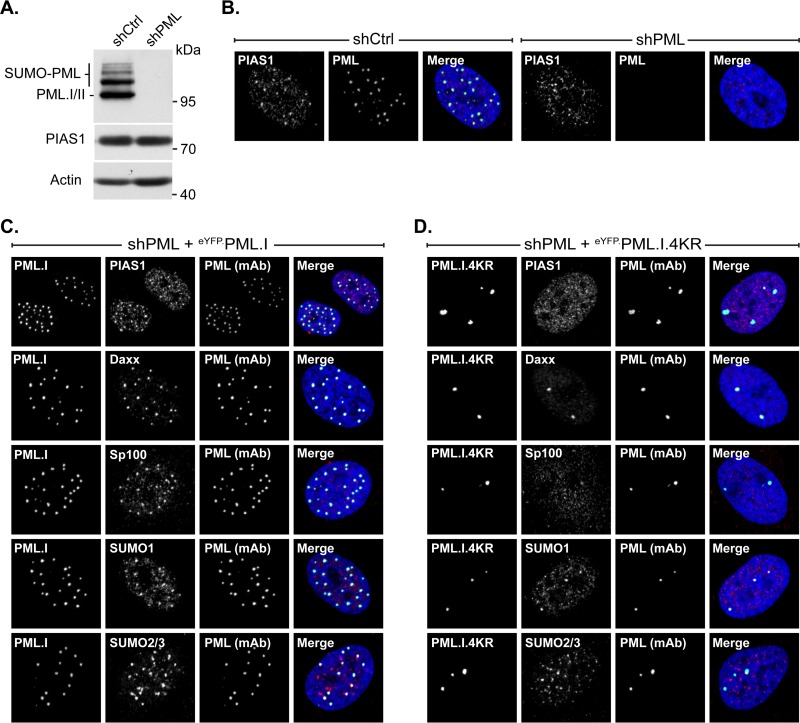
PIAS1 localization at PML-NBs requires SUMO-modified PML. (A) Western blots show PML and PIAS1 protein levels in transgenic cells that express PML-specific shRNA (shPML) or a nontargeted control sequence (shCtrl). Whole-cell lysates were resolved by Tris-glycine SDS-PAGE. Membranes were probed for PML or PIAS1 and for actin as a loading control. Molecular masses are indicated. (B) Confocal microscopy images show the nuclear localization of PIAS1 in transgenic HFt cells that express shPML or shCtrl. PIAS1 (red) and PML (green) were detected by indirect immunofluorescence. (C and D) Confocal microscopy images show PIAS1 localization with respect to PML isoform I in PML-depleted transgenic HFt cells stably reconstituted with PML.I (eYFP.PML.I [C]) or SUMOylation-deficient PML.I (eYFP.PML.I.4KR [D]). PIAS1 (red), Daxx (red), Sp100 (red), SUMO1 (red), SUMO2/3 (red), and PML (cyan) were detected by indirect immunofluorescence. Nuclei were stained with DAPI (blue). mAb, monoclonal antibody.

The localization of PIAS1 at PML-NBs was evaluated in PML-depleted cells stably reconstituted with PML isoform I (eYFP.PML.I) or PML.I with substitution mutations in the four major SUMOylation acceptor sites (eYFP.PML.I.4KR) to test the potential dependency of PIAS1 localization at PML-NBs on PML SUMOylation (5, 10). PML depletion was achieved through the stable transduction of vectors that express PML-specific short hairpin RNA (shRNA) (9, 22). The depletion of PML did not obviously affect PIAS1 expression (Fig. 3A). In the absence of PML, and consequently PML-NBs (7, 8), PIAS1 primarily localized to MARs (Fig. 3B). Reconstitution of PML-depleted cells with eYFP did not stimulate the formation of PML-NBs or otherwise alter PIAS1 subnuclear localization (data not shown), whereas reconstitution with eYFP.PML.I was sufficient to nucleate PML-NB formation and PIAS1 associated with these structures (Fig. 3C). SUMO modification of PML is essential for the proper assembly of PML-NBs (7, 8, 43); therefore, reconstitution with SUMOylation-deficient PML.I resulted in the formation of aberrant aggregates (Fig. 3D) (8, 10). Some PML-NB constituent proteins, such as Daxx and SUMO1, associated with these aggregates, while others, such as Sp100 and SUMO2/3, largely did not (Fig. 3D). The localization of Daxx at PML-NBs is largely mediated through SIM-dependent association with SUMO1; thus, SUMO1 interaction with the PML.I.4KR SIM, or the localization of an alternative SUMO1-modified protein at the PML.I.4KR aggregates, likely mediated this association of Daxx (7, 43). In contrast, PIAS1 did not localize at the aggregates of SUMOylation-deficient PML. These data demonstrate that SUMO-modified, or SUMOylation-competent, PML is required for the stable association of PIAS1 at PML-NBs and that Daxx or SUMO1 is not sufficient to mediate this association in the absence of SUMOylation-competent PML.

### Localization of PIAS1 at nuclear domains that contain infecting HSV-1 genomes is disrupted by ICP0.

**FIG 4:**
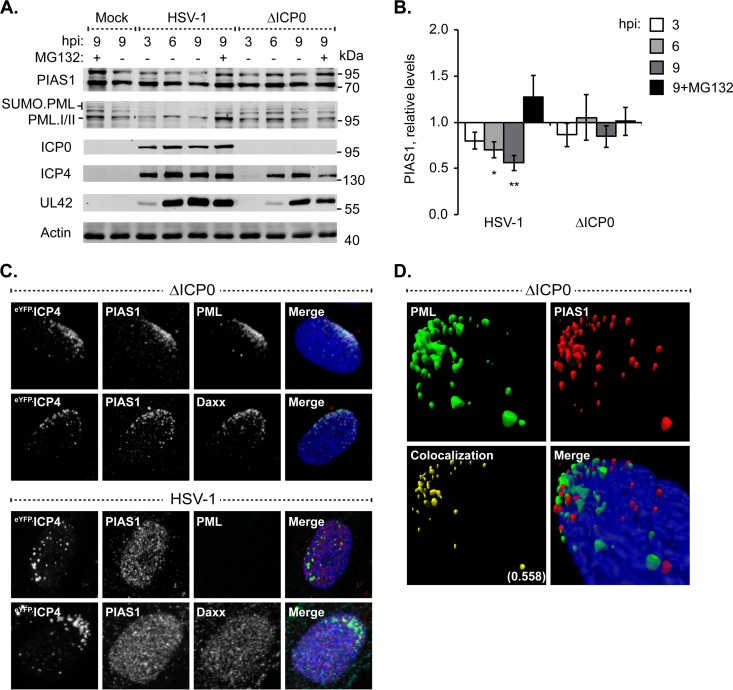
ICP0 disperses PIAS1 away from domains that contain infecting HSV-1 genomes without targeting it for proteasomal degradation. (A) Western blots show PIAS1 protein levels during wild-type or ICP0-null mutant HSV-1 infection. HFt cells were infected with 10 PFU of wild-type (HSV-1) or ICP0-null mutant (ΔICP0) HSV-1 per cell in the presence (+) or absence (−) of the proteasome inhibitor MG132. Whole-cell lysates were harvested at 3, 6, or 9 h postinfection (hpi), and proteins were resolved by Tris-Tricine SDS-PAGE. Membranes were probed for PIAS1, PML as an example of an ICP0 substrate, ICP0, ICP4, and UL42 to show the progression of infection, and actin as a loading control. Molecular masses are indicated. (B) Bar graph shows the average relative levels of PIAS1 during infection with wild-type or ICP0-null mutant HSV-1. The intensities of PIAS1 protein bands were quantitated from Western blots as in panel A, normalized to their respective loading control, and presented as a ratio to the level in mock-infected cells at 9 hpi (1.0). Means and standard error of the means (SEM) are shown (*n* = 7). *, *P* < 0.05; **, *P* < 0.01 (Student's two-tailed *t* test). (C) Confocal microscopy images show the accumulation of PIAS1, or lack of it, at the nuclear edge associated with HSV-1 genome entry in cells within the periphery of a developing plaque (20). HFt cells were infected with 2 or 0.002 PFU per cell of ICP0-null mutant or wild-type HSV-1, respectively, for 16 h. PIAS1 (red), PML (cyan), and Daxx (cyan) were detected by indirect immunofluorescence. The nuclear edge associated with HSV-1 genome entry was identified by eYFP.ICP4 localization. Nuclei were stained with DAPI (blue). (D) Three-dimensional reconstruction of z-stack series of confocal microscopy images shows the accumulation of PIAS1 and PML at the nuclear edge associated with HSV-1 genome entry. Regions of PML and PIAS1 colocalization (yellow) are highlighted; the Pearson coefficient is indicated.

ICP0 counteracts PML-NB-mediated intrinsic immunity by targeting specific antiviral proteins for proteasome-dependent degradation or dispersal away from infecting viral genome foci (reviewed in reference 25). Therefore, prior to investigation into the potential roles of PIAS1 in intrinsic immunity, PIAS1 protein levels were evaluated during HSV-1 infection to ascertain whether it is a substrate for ICP0-mediated degradation. HFt cells infected with wild-type HSV-1 had a continued decrease in PIAS1 protein levels as infection progressed, down to 56% ± 9% of the levels in mock-infected cells by 9 h postinfection (hpi) (Fig. 4A and B; *P* ≤ 0.01). In contrast, PML, which is a direct target of ICP0, had a substantial loss (typically 50 to 70%) in protein levels as early as 3 hpi (Fig. 4A). In the absence of ICP0, a significant loss in PIAS1 protein levels was not observed (Fig. 4A and B). Given the kinetics of PIAS1 degradation and the lack of direct interaction with ICP0 (45), PIAS1 is not likely a substrate for ICP0-targetted proteasomal degradation.

The relocalization of PML-NB constituent proteins from punctate PML-NBs throughout the nucleus to a nuclear edge associated with HSV-1 genome entry is characteristic of the PML-NB-mediated intrinsic immune response (for example, see Fig. 4C, PML, Daxx, ΔICP0) (10, 20). During infection with ICP0-null mutant HSV-1, PIAS1 also relocalized to the nuclear edge that contained infecting viral genomes, which was identified by the localization of PML or Daxx and the HSV-1 DNA-binding protein ICP4 (Fig. 4C, ΔICP0). Reconstruction of a z-stack series of confocal microscopy images demonstrated that PIAS1 and PML colocalized at the nuclear edge associated with viral genome entry (Pearson coefficient, 0.558), although foci that predominantly contained only PML or PIAS1 were also evident (Fig. 4D). The relocalization of PIAS1 to nuclear domains that contained infecting HSV-1 genomes was disrupted during infection with wild-type HSV-1 (Fig. 4C, HSV-1). Under these infection conditions, PIAS1 had a nuclear diffuse distribution similar to that of Daxx (Fig. 4C, HSV-1).

These data demonstrate that PIAS1 is recruited to nuclear domains that contain infecting HSV-1 genomes in a manner that is disrupted by ICP0 through mechanisms that do not directly target PIAS1 for degradation.

### PML enhances PIAS1 SIM-dependent localization at nuclear domains that contain infecting HSV-1 genomes.

**FIG 5:**
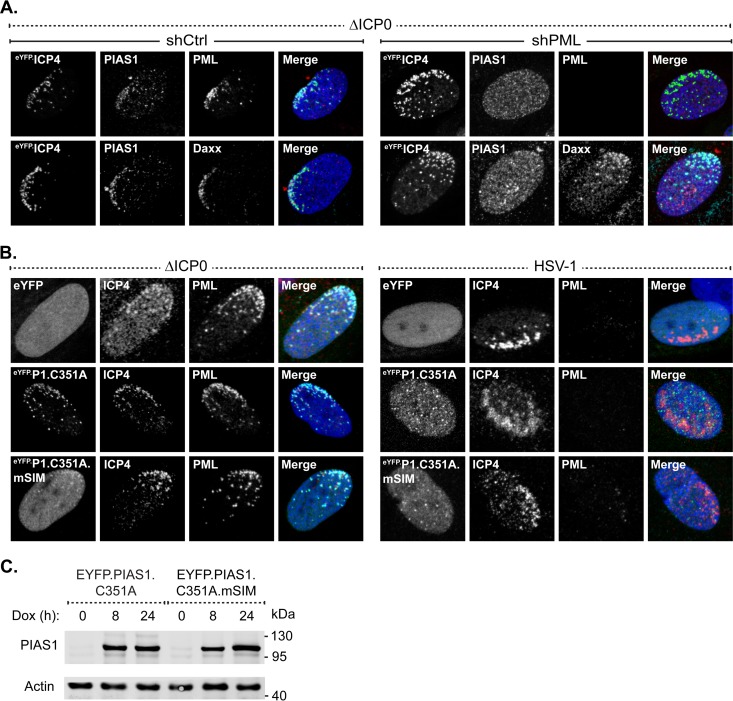
The SIM-dependent localization of PIAS1 at domains that contain infecting HSV-1 genomes is enhanced by PML. (A) Confocal microscopy images show PIAS1 localization, or lack of it, at nuclear domains that contain infecting HSV-1 genomes in transgenic PML depleted (shPML) or not (shCtrl) HFt cells within a developing plaque. Cells were infected with 2 PFU of ICP0-null mutant HSV-1 (ΔICP0) per cell for 16 h. PIAS1 (red), PML (cyan), and Daxx (cyan) were detected by indirect immunofluorescence. (B) Confocal microscopy images show the nuclear localization of eYFP or eYFP-PIAS1 mutant proteins in HSV-1-infected cells within a developing plaque. EYFP or eYFP-PIAS1 mutant protein expression was DOX induced for 4 h prior to infection with 2 PFU of ICP0-null mutant HSV-1 (ΔICP0) or 0.002 PFU of wild-type HSV-1 (HSV-1) per cell for 16 h. ICP4 (red) and PML (cyan) were detected by indirect immunofluorescence. The nuclear edge associated with HSV-1 genome entry is identified by ICP4 localization. Nuclei were stained with DAPI (blue). (C) Western blots show the expression level of eYFP-PIAS1 mutant proteins. Protein expression was DOX induced for 0, 8, or 24 h prior to the collection of whole-cell lysates. Membranes were probed for PIAS1 and actin as a loading control. Molecular masses are indicated.

Constituent PML-NB antiviral proteins are individually recruited to nuclear domains that contain infecting HSV-1 genomes through SUMO-dependent mechanisms that are independent of PML (10, 20, 22). To initiate characterization of the recruitment mechanisms of PIAS1, its recruitment phenotype was evaluated in PML-depleted cells. Robust depletion of PML was achieved through the stable transduction of plasmids that express PML-specific shRNA (Fig. 3A and B and 5A) (22). As expected, Daxx was recruited to the nuclear edge associated with ICP0-null mutant HSV-1 genome entry independently of PML (Fig. 5A) (10, 22). In contrast, PIAS1 recruitment was significantly diminished in the absence of PML (Fig. 5A), suggesting that this process is largely PML dependent. PIAS1 was still stably recruited to nuclear domains that contained infecting viral genomes in cells that expressed nontargeted control shRNAs, confirming that the lack of recruitment was due to PML depletion and not transduction or the selection of transgenic cells (Fig. 5A). These data demonstrate that PML promotes the stable accumulation of PIAS1 in nuclear domains that contain infecting HSV-1 genomes.

Thus far, PIAS1 represents the first PML-NB constituent protein that is recruited to domains that contain infecting HSV-1 genomes through mechanisms that are enhanced by PML. As the association of PIAS1 with PML-NBs is SIM dependent, the role of the SIM in the recruitment mechanism of PIAS1 was evaluated. Mutation of the SIM substantially reduced PIAS1 accumulation at the nuclear edge that contained infecting ICP0-null mutant HSV-1 genomes (Fig. 5B, ΔICP0, bottom row). The reduced accumulation of mutant PIAS1 was not due to the inactivation of its catalytic activity, as the inactive PIAS1 mutant with a functional SIM still stably accumulated in nuclear domains that contained infecting viral genomes (Fig. 5B, ΔICP0, middle row). As the expression levels of the mutant PIAS1 proteins were similar (Fig. 5C), the reduced accumulation of the SIM mutant was not due to overexpression saturating potential binding sites within the nuclear domains that contained infecting viral genomes. As expected, ICP0-mediated degradation of PML abrogated the recruitment of PIAS1 to the nuclear periphery associated with infecting HSV-1 genomes regardless of any mutations to PIAS1 (Fig. 5B, HSV-1).

These data demonstrate that PIAS1 is recruited to nuclear domains that contain infecting HSV-1 genomes through SIM-dependent mechanisms that are enhanced by PML. Furthermore, the stable localization of PIAS1 at the nuclear edge associated with HSV-1 genome entry is likely independent of PIAS1 catalytic activity.

### PIAS1 is not essential for PML SUMO modification or the formation of PML-NBs.

**FIG 6:**
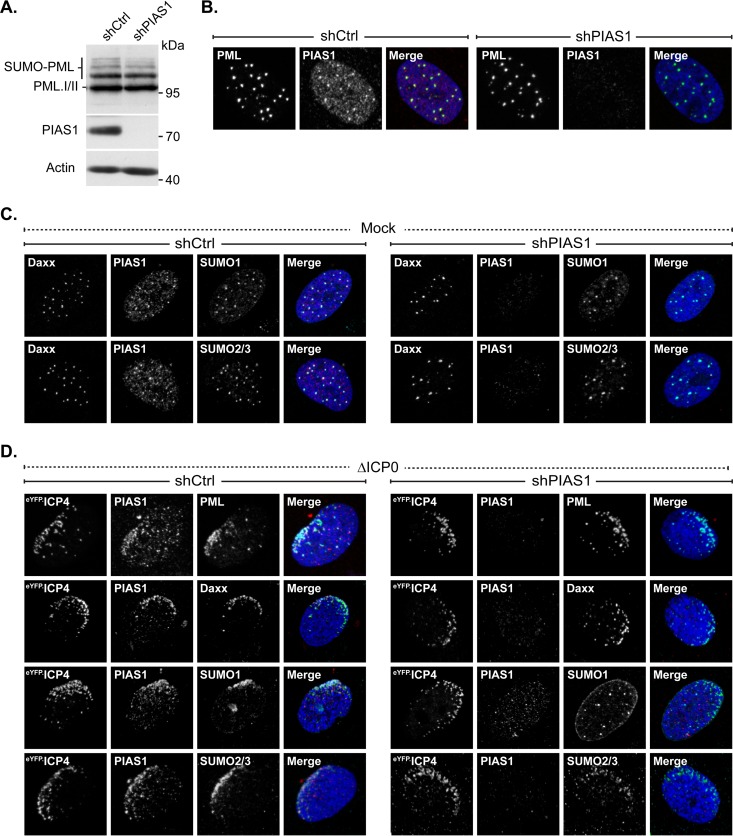
PIAS1 is not essential for PML-NB formation or PML SUMOylation but does enhance the accumulation of SUMO1 in domains that contain infecting HSV-1 genomes. (A) Western blots show PIAS1 protein levels in transgenic HFt cells that express PIAS1-specific (shPIAS1) or nontargeted control (shCtrl) shRNA. Whole-cell lysates were resolved by Tris-glycine SDS-PAGE. Membranes were probed for PIAS1 or PML and for actin as a loading control. Molecular masses are indicated. (B) Confocal microscopy images show the localization of PML in transgenic shPIAS1- or shCtrl-expressing cells. PML (green) and PIAS1 (red) were detected by indirect immunofluorescence. (C) Confocal microscopy images show the localization of Daxx, SUMO1, or SUMO2/3 in mock-infected HFt cells that express shPIAS1 or shCtrl. Daxx (green), PIAS1 (red), SUMO1 (cyan), and SUMO2/3 (cyan) were detected by indirect immunofluorescence. (D) Confocal microscopy images show the nuclear localization of PML, Daxx, SUMO1, or SUMO2/3 during ICP0-null mutant HSV-1 infection of transgenic HFt cells that express shPIAS1 or shCtrl. Cells were infected with 2 PFU of ICP0-null mutant HSV-1 per cell for 16 h. PIAS1 (red), PML (cyan), Daxx (cyan), SUMO1 (cyan), and SUMO2/3 (cyan) were detected by indirect immunofluorescence. The nuclear edge associated with HSV-1 genome entry was identified by eYFP.ICP4 localization. Nuclei were stained with DAPI (blue).

The potential functional significance of PIAS1 to regulate the association of select PML-NB constituent proteins at PML-NBs or their stable accumulation in nuclear domains that contain infecting HSV-1 genomes was tested in PIAS1-depleted cells. Transgenic HFt cells that expressed PIAS1-specific shRNA had a marked reduction in PIAS1 expression relative to that of cells that expressed nontargeted control shRNA (Fig. 6A). After limited passaging, however, PIAS1 protein expression recovered to levels similar to those in transgenic cells that expressed control shRNA (data not shown). Experiments were therefore conducted over multiple rounds of independent transduction with minimal passaging of the isolated transgenic cells. The depletion of PIAS1 did not noticeably alter PML expression or SUMO modification (Fig. 6A) (31). Furthermore, the association of the major constituent PML-NB proteins Daxx, SUMO1, or SUMO2/3 at PML-NBs, or the relative size and number of PML-NBs per nucleus, was not noticeably altered following PIAS1 depletion (Fig. 6B and C).

PML, Daxx, or SUMO2/3 still localized to sites associated with infecting ICP0-null mutant HSV-1 genomes in PIAS1-depleted cells (Fig. 6D), demonstrating that PIAS1 is not necessary for their stable accumulation in these domains. In contrast, the accumulation of SUMO1 at the nuclear edge associated with viral genome entry was notably reduced in PIAS1-depleted cells (Fig. 6D), highlighting a role for PIAS1 in the stable accumulation of SUMO1 at these sites. As expected, ICP0 efficiently disrupted the localization of constituent PML-NB antiviral proteins at nuclear domains that contained infecting HSV-1 genomes regardless of the presence or absence of PIAS1 (data not shown).

These data demonstrate that PIAS1 is not essential for PML SUMOylation or the proper formation of PML-NBs. During infection with ICP0-null mutant HSV-1, PIAS1 is differentially required for the stable localization of constituent PML-NB proteins at nuclear domains that contain infecting viral genomes. The stable accumulation of SUMO1 at such domains is enhanced by PIAS1, whereas PML, SUMO2/3, and Daxx effectively relocalize to such domains independently of PIAS1.

### PIAS1 contributes to the cellular restriction of ICP0-null mutant HSV-1.

**FIG 7:**
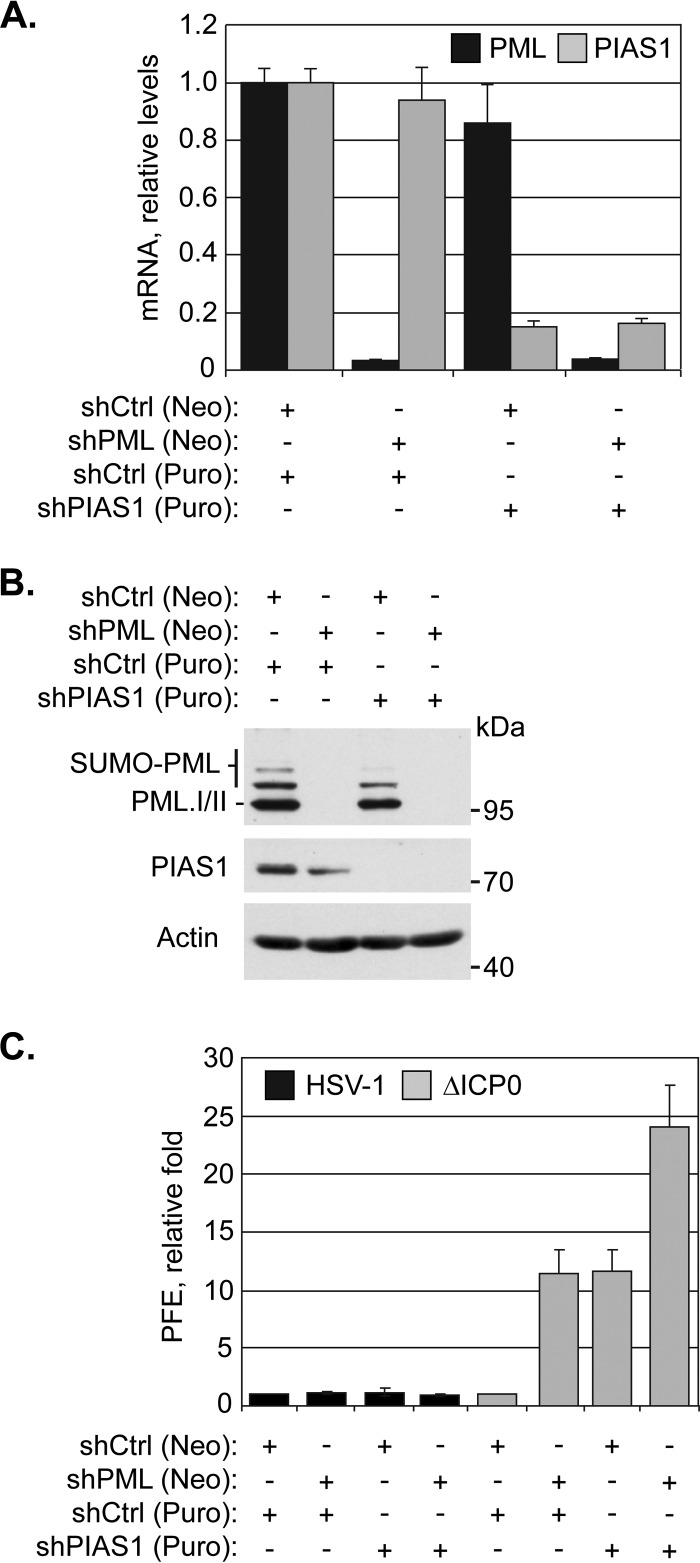
PIAS1 repression of ICP0-null mutant HSV-1 replication is additive to that of PML. (A) Bar graph showing the average relative levels of PML or PIAS1 mRNA in transgenic HFt cells that express shRNA against PML (shPML), PIAS1 (shPIAS1), or a nontargeted control (shCtrl). PML or PIAS1 mRNA levels were determined using the TaqMan system of quantitative RT-PCR. Values normalized to GAPDH expression using the threshold cycle (ΔΔ*C_T_*) method are expressed relative to mock-infected cells (1.0). Means and standard deviations (SD) are shown (*n* > 3). (B) Western blots show PML or PIAS1 protein levels in transgenic HFt cells that express shPML, shPIAS1, or shCtrl. Whole-cell lysates were resolved by Tris-glycine SDS-PAGE. Membranes were probed for PML or PIAS1 and for actin as a loading control. Molecular masses are indicated. (C) Bar graph showing the average relative plaque forming efficiency (PFE) of wild-type (HSV-1) or ICP0-null mutant (ΔICP0) HSV-1 in transgenic HFt cells that express shCtrl, shPML, or shPIAS1. The number of plaques for each strain is expressed relative to the corresponding number of plaques for that strain in shCtrl-expressing cells. Means and SD are shown (*n* = 6). Neo, neomycin; Puro, puromycin.

To test the functional significance of PIAS1 during infection, the replication of wild-type or ICP0-null mutant HSV-1 was evaluated in cells depleted of PIAS1 or of PML as a positive control (22). Transgenic HFt cells that expressed PIAS1- or PML-specific shRNA, alone or in combination, had greater than 80% depletion in the specific target mRNA and a substantial reduction in the corresponding protein levels relative to cells that expressed nontargeted control shRNA (Fig. 7A and B). The depletion of PIAS1 did not substantially alter the plaque forming efficiency (PFE) of wild-type HSV-1, demonstrating that PIAS1 is not essential for HSV-1 replication (Fig. 7C). In contrast, the PFE of ICP0-null mutant HSV-1 was enhanced 10-fold in PIAS1-depleted cells, which was comparable to the increase observed following PML depletion (Fig. 7C). PIAS1 is thus identified as an intrinsic antiviral factor that contributes to the intracellular restriction of ICP0-null mutant HSV-1.

As the depletion of PIAS1 and PML resulted in similar levels of relief of restriction in ICP0-null mutant HSV-1 replication, and PML facilitates the stable accumulation of PIAS1 in domains that contain infecting HSV-1 genomes, it is possible that the mechanisms of PIAS1- and PML-mediated viral restriction are sequential. To test this possibility, HSV-1 replication was evaluated in cells depleted of both PML and PIAS1 (Fig. 7A and B). In the absence of PIAS1 and PML, the PFE of ICP0-null mutant HSV-1 was twice that in cells depleted of either protein alone (Fig. 7C). These data suggest that the antiviral mechanisms of PIAS1 and PML are likely additive. Under conditions in which PIAS1 and PML were both depleted, the replication of wild-type HSV-1 was not substantially affected, confirming that neither protein is essential for viral replication and that ICP0 is sufficient to counteract their intrinsic antiviral activities (Fig. 7C).

### The intrinsic antiviral mechanisms of PIAS1 and PIAS4 are cooperative.

**FIG 8:**
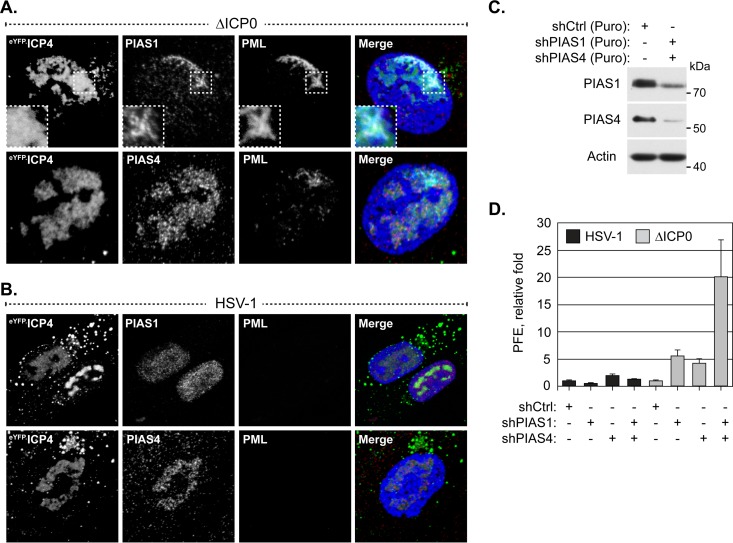
PIAS1 and PIAS4 cooperatively restrict ICP0-null mutant HSV-1 replication. (A and B) Confocal microscopy images show the localization of PIAS1 and PIAS4 in HSV-1-infected cells. HFt cells were infected with 2 or 0.002 PFU per cell of ICP0-null mutant (A) or wild-type HSV-1 (B), respectively, for 16 h. PIAS1 (red), PIAS4 (red), and PML (cyan) were visualized by indirect immunofluorescence. Replication compartments were identified by the accumulation of eYFP.ICP4. Insets (dashed boxes in panel A) highlight a region where PIAS1 and PML colocalize within a replication compartment. Nuclei were stained with DAPI (blue). (C) Western blots show PIAS1 or PIAS4 protein levels in transgenic HFt cells that express shRNA against PIAS1 (shPIAS1), PIAS4 (shPIAS4), or a nontargeted control (shCtrl). Whole-cell lysates were resolved by Tris-glycine SDS-PAGE. Membranes were probed for PIAS1 or PIAS4 and for actin as a loading control. Molecular masses are indicated. Puro, puromycin. (D) Bar graph showing the average relative PFE of wild-type or ICP0-null mutant HSV-1 in transgenic HFt cells that express shPIAS1, shPIAS4, or shCtrl. The number of plaques for each strain is expressed relative to the corresponding number of plaques for that strain in cells that express shCtrl. Means and SD are shown (*n* = 6).

The identification of PIAS1 as an intrinsic antiviral PML-NB constituent protein complements the recent identification of PIAS4 as an intrinsic antiviral factor (30). As PIAS4 localizes to domains that contain HSV-1 genomes throughout infection (30), whether or not PIAS1 also accumulates in replication compartments was evaluated. During infection with ICP0-null mutant HSV-1, PIAS1 localized to replication compartments, although this localization phenotype was less prominent than that of PIAS4 (Fig. 8A). Moreover, the localization of PIAS1 in HSV-1 replication compartments was disrupted by ICP0 (Fig. 8B), whereas PIAS4 remained associated with replication compartments irrespective of ICP0 expression (Fig. 8B) (30).

To test the functional relationship between the antiviral activities of PIAS1 and PIAS4, HSV-1 replication was evaluated in cells depleted of both proteins. Transgenic HFt cells that expressed PIAS1 or PIAS4 specific shRNA had considerable, although not full, depletion of either protein (Fig. 8C). Depletion of PIAS1 and PIAS4 alone or in combination did not substantially alter the replication of wild-type HSV-1, confirming that ICP0 is sufficient to counteract their antiviral activities and that these proteins are not essential for HSV-1 replication (Fig. 8D). Depletion of PIAS1 and PIAS4 alone enhanced the replication of ICP0-null mutant HSV-1 to similar degrees, while depletion of both enhanced it to an even greater degree than did depletion of either protein alone (Fig. 8D). PIAS1 and PIAS4 therefore likely mediate the restriction of ICP0-null mutant HSV-1 through cooperative but independent mechanisms, suggesting that the complementary functional roles of PIAS proteins in the regulation of innate immunity are mirrored in their regulation of intrinsic immunity.

## DISCUSSION

We identify PIAS1 as a PML-NB constituent protein that contributes to the repression of ICP0-null mutant HSV-1 replication mediated by the intrinsic antiviral immune response. PIAS1 associates with PML-NBs through SIM-dependent mechanisms that require SUMOylation-competent PML and is recruited to nuclear domains that contain infecting HSV-1 genomes through SIM-dependent mechanisms that are enhanced by PML. The stable accumulation of SUMO1 at domains that contain infecting HSV-1 genomes is enhanced by PIAS1, while the stable localization of other major PML-NB constituent proteins at these domains is largely independent of PIAS1. The antiviral mechanisms of PIAS1 are additive to those of PML and cooperative with those of PIAS4. However, the antiviral activities of PIAS1 are efficiently counteracted by ICP0, which abrogates the stable accumulation of PIAS1 in nuclear domains that contain viral genomes. Similar to that of Daxx, the antagonism of PIAS1 occurs without directly targeting it for degradation (32).

PIAS1 is unique among the PIAS family of SUMO ligases in that it is the only member that substantially associates with PML-NBs in HFt cells (Fig. 1) (30). This localization of PIAS1 was not restricted to HFt cells, however, as it also colocalized with PML-NBs in other restrictive cell types (Fig. 1C). Given that the PIAS family of proteins have highly conserved SIMs (80% amino acid identity), the SIM-mediated association of PIAS1 with PML-NBs is likely contextual rather than due to generic interactions with SUMO moieties. PML.I.4KR aggregates contain SUMO1 and, to a much lesser degree, SUMO2/3 (Fig. 3D). In this context, SUMO1 is not sufficient to facilitate the stable localization of PIAS1 at the PML.I.4KR aggregates. Even though the SIM (VIDLT) of PIAS1 does not preferentially bind to a particular SUMO isoform (46, 47), peptides that contain the core SIM sequence (I/V)DLT preferentially bind to SUMO2/3 (48). PIAS1 localization at PML-NBs could therefore be largely mediated through SUMO2/3 interactions. Alternatively, but not exclusively so, specific SUMOylated proteins, potentially PML, may regulate PIAS1 localization at PML-NBs. The association of Sp100 with PML-NBs is not mediated by the Sp100 SIM (49), which superficially suggests that Sp100 should localize to the PML.I.4KR aggregates (Fig. 3D). However, the localization of Sp100 at PML-NBs is mediated by MORC3 (50–52), which does associate with PML-NBs through SIM-dependent mechanisms (53). Sp100 association with PML-NBs is thus indirectly mediated through the SIM of MORC3. PIAS1-specific interactions with particular SUMOylated proteins within PML-NBs may therefore provide the specificity for the stable association of PIAS1, but not other PIAS family members, at these structures.

The mechanism whereby PIAS1 is recruited to nuclear domains that contain infecting HSV-1 genomes is similar to that of other PML-NB constituent proteins in that it is SIM dependent, but it is also unique in that it is enhanced by PML. In the absence of PML, the localization of PIAS1 at the nuclear edge associated with viral genome entry is substantially reduced (Fig. 5A), indicating that PML is required for the efficient or stable accumulation of PIAS1 at these sites. However, PIAS1 and PML do not fully colocalize within these nuclear domains, and puncta that primarily contain only PIAS1 or PML are evident (Fig. 4D). These data suggest that PIAS1 can stably remain within domains that contain infecting HSV-1 genomes through interactions that are likely independent of PML; however, such interactions are not sufficient to mediate its stable relocalization in the absence of PML. Although the accumulation of PIAS1 in nuclear domains that contain infecting HSV-1 genomes is largely PML dependent, the antiviral effects of PIAS1 are additive to those of PML (Fig. 7C), which demonstrates that they have separate antiviral mechanisms. Thus, in the absence of PML, and consequently the stable accumulation of PIAS1 in nuclear domains that contain infecting viral genomes, PIAS1 retains antiviral activities. Conversely, the antiviral mechanisms of PML are not exclusively linked to its capacity to recruit PIAS1 to nuclear domains that contain infecting HSV-1 genomes.

It has been reported that the accumulation of SUMO1 within nuclear domains that contain infecting HSV-1 genomes represents SUMOylated PML, as conditions that limit the recruitment of PML also decrease the accumulation of SUMO1 at the recruitment edge (10, 21, 24). However, we now show that in the absence of PML, the lack of SUMO1 accumulation at the recruitment edge is likely a secondary effect of the inefficient recruitment of PIAS1 (Fig. 5A and 6D). PIAS1 may promote the accumulation of SUMO1 at the recruitment edge through direct SUMOylation of target substrates. However, preferential PIAS1-mediated SUMO1 modification rather than SUMO2/3 modification of target substrates has yet to be demonstrated. Alternatively, PIAS1 could promote the accumulation of SUMO1 at the recruitment edge through interactions with specific SUMO1-modified proteins. It is unlikely, however, that the efficient accumulation of SUMO1 in nuclear domains that contain infecting HSV-1 genomes represents the primary mechanism of PIAS1 antiviral activity. The depletion of PML disrupts PIAS1, and consequently SUMO1, accumulation in nuclear domains that contain infecting HSV-1 genomes. The accumulation of SUMO1 at such domains is therefore impaired in the absence of either PML or PIAS1, whereas the depletion of both proteins impairs the intrinsic immune response to a greater degree than the depletion of either protein alone (Fig. 7C).

PIAS1 is the only PIAS protein that substantially associates with PML-NBs in HFt cells (Fig. 1). However, PML-NB formation, PML SUMOylation, or the localization of SUMO1 or -2/3 within PML-NBs is not obviously altered in PIAS1-depleted cells (Fig. 6). This observation suggests that PIAS1 is largely not responsible for the SUMOylation events that are crucial for PML-NB stabilization, constitutive PML SUMOylation, or the localization of SUMO1 or -2/3 at PML-NBs. Alternatively, other PIAS family members (or SUMO E3 ligases) may mediate these events under conditions of PIAS1 depletion, or it is possible that the PIAS1 at PML-NBs largely functions to regulate PML turnover (31). However, the latter scenario is unlikely, as we did not observe any significant accumulation of unmodified PML following the depletion of PIAS1 (Fig. 6A). During infection with ICP0-null mutant HSV-1, the depletion of PIAS1 dramatically reduces SUMO1 accumulation at the nuclear edge that contains infecting HSV-1 genomes (Fig. 6D), suggesting that other SUMO E3 ligases do not efficiently mediate this particular PIAS1 function. PIAS1 and PIAS4 are currently the only PIAS proteins identified to be intrinsic antiviral factors (30; this study). While both proteins localize to nuclear domains that contain viral DNA throughout infection, PIAS1 is more prominently recruited to domains that contain infecting HSV-1 genomes, while PIAS4 is more prominently recruited into replication compartments (Fig. 8A and B) (30). The localization phenotypes of PIAS1 and PIAS4 during HSV-1 infection suggest that they have shared as well as distinct antiviral activities. Consistently, the antiviral mechanisms of PIAS1 and PIAS4 are cooperative (Fig. 8D). Moreover, PIAS1 and PIAS4 both complement the antiviral activities of PML (Fig. 7C) (30). Together, these data indicate that PIAS proteins likely utilize a multifaceted yet interconnected approach to modulate intrinsic immunity to achieve the ultimate objective of viral repression.
